# Exclusive Breastfeeding and Factors Influencing Its Abandonment During the 1st Month Postpartum Among Women From Semi-rural Communities in Southeast Mexico

**DOI:** 10.3389/fped.2022.826295

**Published:** 2022-02-18

**Authors:** Inocente Manuel Vázquez-Osorio, Rodrigo Vega-Sánchez, Eric Maas-Mendoza, Solange Heller Rouassant, María Eugenia Flores-Quijano

**Affiliations:** ^1^Licenciatura de Nutrición, División Académica de Ciencias de la Salud, Universidad Juárez Autónoma de Tabasco, Villahermosa, Mexico; ^2^Jurisdicción Sanitaria 4 del Municipio de Centro, Secretaría de Salud, Villahermosa, Mexico; ^3^Departamento de Nutrición y Bioprogramación, Instituto Nacional de Perinatología, Mexico City, Mexico; ^4^Private Practitioner, Naucalpan, Estado de México, Mexico, Mexico

**Keywords:** exclusive breastfeeding (EBF), breastfeeding, infant feeding, Mexico, Tabasco (Mexico), food insecurity, social determinants of health, breastfeeding beliefs

## Abstract

**Introduction:**

In this study we describe breastfeeding practices among women from semi-rural communities in southeast Mexico, and explore which factors, modifiable or not, are associated with such practices.

**Materials and Methods:**

This was a formative cross-sectional study that included 143 mothers with infants 4–6 months old, from semi-rural communities in Tabasco, Mexico. We collected data on two categories of factors: (1) women's sociodemographic characteristics, and (2) maternal / infant factors. We first analyzed the frequency of various breastfeeding practices. Then, we classified participants into the up to 1 month of exclusive breastfeeding group ( ≤ 1 m-EBF) and the beyond 1 month EBF group (>1 m-EBF), if they practiced EBF for less or more than 1 month, respectively. We compared the two categories of factors between groups and then, using logistic regression models, explored which factors were associated with practicing >1 m-EBF.

**Results:**

By the end of the 1st month postpartum, 51.7% of participants had abandoned EBF, introduced milk formula (35%), other food (9.1%), non-nutritive liquids (7.7%), or had stopped breastfeeding completely. In the next months, EBF practice fell sharply and mixed feeding grew importantly.

Logistic regression models showed that women were more likely to be in the >1 m-EBF group if they lived with the baby's father, had complications during pregnancy, delivered vaginally and attended a health center at least three times postpartum. To the contrary, women were less likely to be practice >1 m-EBF if they gave infants other liquids during their hospital stay; experienced pain or discomfort in breasts/nipples, or used a pacifier after hospitalization; had larger bodies (i.e., higher BMI); and believed that you should give the infant powdered milk or some other food when the baby is not full.

**Conclusion:**

Many factors associated with abandoning EBF, particularly in the early postpartum period, are modifiable and can be altered through timely interventions that include giving correct information and ensuring its comprehension; assertive personal counseling and accompaniment must be provided to mothers; and reinforcement during the early postpartum at health facilities and other settings.

## Introduction

Breastfeeding confers life-long benefits to the infant, such as increased likelihood of survival, better health, development, and cognitive achievements (1), which in time contribute to the society's human capital and sustainable development (2). To warrant these benefits, exclusive breastfeeding (EBF) is recommended by the World Health Organization and UNICEF as the optimal way to feed infants for the first 6 months, which means that no other foods or liquids, including water, are provided to them during that period.

In Mexico, data from the latest nationwide surveys show an increase in the prevalence of EBF among infants younger than 6 months, from 13.0 to 20.7% between 2009 and 2018 (3). However, these figures are still below 44%, the global rate of EBF and far from the global target goal of 70%, proposed by the World Health Assembly to be reached by 2030 (4).

International agencies have identified the type of actions that are needed to enable women to breastfeed adequately for an appropriate duration, while initiatives and programs have been proposed to achieve their execution (4). Some of those actions have been undertaken in Mexico by the government, civil society and academia (5). For example, several hospitals offering maternity services have been nominated as “baby friendly;” some of the provisions of the Code of Marketing of Breastmilk Substitutes are contemplated in the Mexican legislation; and a nationwide breastfeeding training program for health service providers was developed. However, there has not been a formal assessment of the effectiveness of these interventions, and some have no national coverage, adequate funding or legislative backing (6).

Moreover, breastfeeding practices may be influenced by many factors of diverse nature, ranging from sociocultural and economic characteristics, to family or social support networks, availability of health services, and mother's attitudes, beliefs or even exposure to breastmilk substitute advertisements (7, 8). Considering such diversity of factors influencing breastfeeding practices, in this study we designed a formative research to (1) describe breastfeeding practices among women from semi-rural communities in Tabasco, southeast Mexico; and (2) to explore which factors condition such practices.

## Materials and Methods

### Study Setting and Design

**Figure 1:**
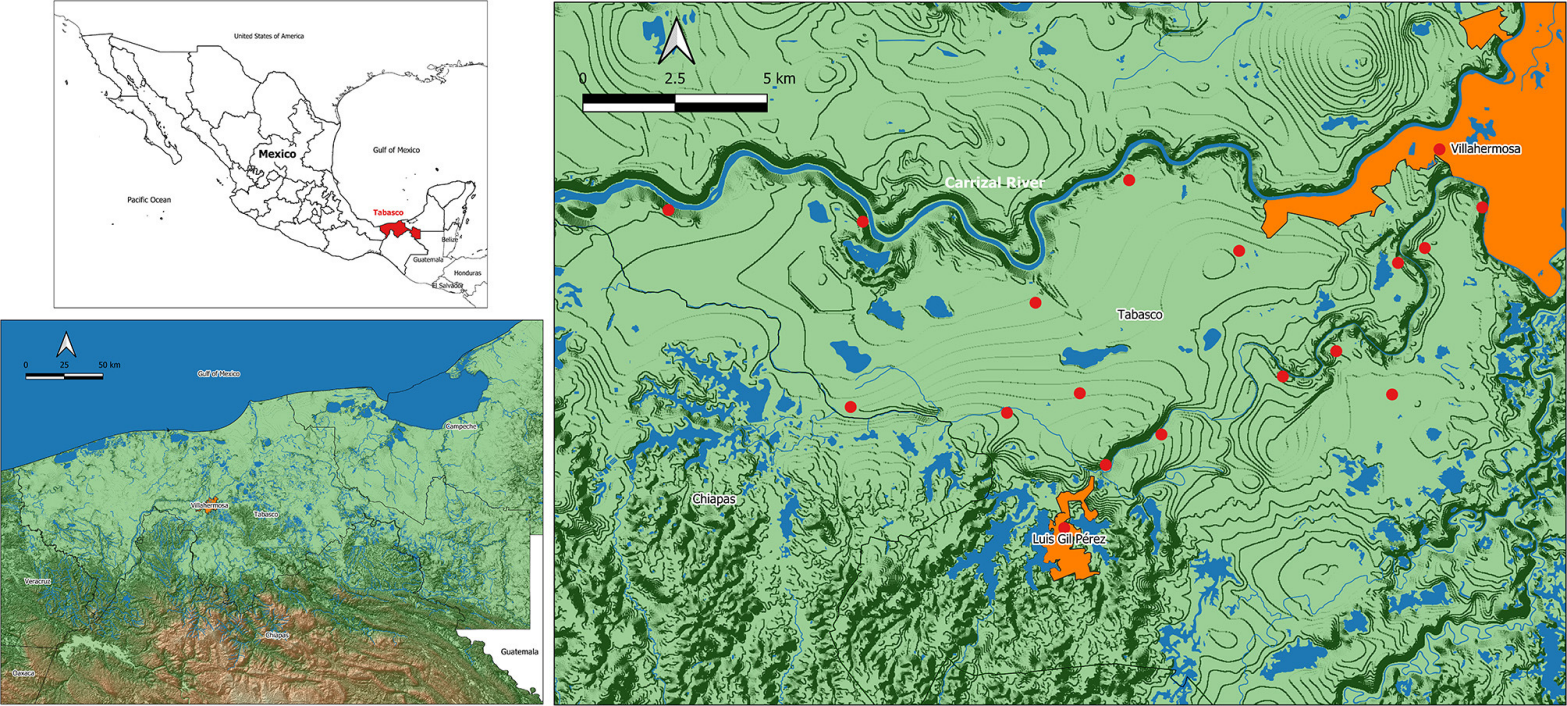
Geographical location of the area of study. Tabasco, in southeast Mexico (top left), is a coastal plain with hot, humid climate and a large presence of wetlands and rivers (bottom left). Participant women received their prenatal medical care in one of the 17 rural first level public health centers (right, red dots) managed from Villa Luis Gil Pérez (orange area to the south), a town located a few kilometers southwest of the state's capital city, Villahermosa (top right corner).

This was a formative, cross-sectional study carried out in Tabasco, a coastal southeastern state in Mexico, characterized by a hot and humid climate and a large presence of rainforests and water bodies (wetlands and rivers) (Figure 1). At the time the study took place (2016), 50.9% of Tabasco's population lived in poverty, of which 11.8% was extreme; these figures have increased since then to 53.6 and 12.3% (9).

The study took place in Centro, one of Tabasco's 17 municipalities/health jurisdictions, and included women who received prenatal care at the Health Center with Expanded Services (CESSA, initials in Spanish), located in an urban town called Villa Luis Gil Pérez, or at one of the 17 first level public health units (FLPHU) affiliated to CESSA. The FLPHU are smaller health centers located in rural or semi-rural villages (Figure 1). Most of the CESSA and FLPHU users are people with the most basic governmental social security (Seguro Popular) and often among low socioeconomic levels.

The study's protocol was approved by the Research and Ethics Committees of the National Institute of Perinatology in Mexico City (212250-3310-11406-03-16) and authorized by the local health authorities at Centro Health Jurisdiction in Tabasco. Data was collected from March to June 2016.

We included women and their babies if they (1) lived within the geographical limits of Villa Luis Gil Pérez, (2) received prenatal care at CESSA or one of the 17 health units, (3) had a single and clinically healthy pregnancy, (4) had not been hospitalized for any condition that could be a barrier for breastfeeding initiation; (5) babies were between 4 and 6 months old at the time of the study, and (6) accepted to participate and signed an informed consent.

We identified potential participants from a census of women who carried out their prenatal control in Villa Luis Gil Pérez's CESSA. We invited these women to participate in the study by telephone and / or by home visits. Data was collected between March and July 2016.

### Sample Size

The sample size was calculated to estimate the proportion of women that would be breastfeeding exclusively in our study population. We considered a precision of 5%, a confidence level of 95%, and the prevalence of exclusive breastfeeding in children under 6 months available at the time: 14.4% nationwide and 15.5% for the southern states of the country (10). With these parameters, the sample size initially considered was 190–200 women. However, due to the much lower exclusive breastfeeding prevalence we found in the community during the course of the study, the aimed sample size was modified to 150 women.

We evaluated selection bias by comparing the basic sociodemographic data from included and not included women. This data was collected by applying a general characteristics questionnaire when inviting women to participate (Supplementary Material Section A).

### Study Variables and Data Analysis

For this study, we included a series of variables related to the participant's characteristics, experiences, and thoughts. Data for constructing these variables was obtained from an *ad-hoc* questionnaire that we applied to participants during the study appointment (Supplementary Material). Data were either used as reported, collapsed and/or developed into categories as a means of data reduction.

Since infants 4–6 months old were included in our study, it was not possible to use the status quo “exclusive breastfeeding under 6 months” indicator proposed by the WHO which considers infant feeding current practices (i.e., previous day). Moreover, we think the WHO status quo EBF indicator has one major limitation: if the mother is only asked what her child ate the day before, it is possible that in previous days the infant ate or drank something other than breast milk. This food would not be registered and therefore lead to an overestimation of exclusive breastfeeding figures.

Therefore, in order to be able to describe the moment when EBF was stopped, we asked participant women their infant's age in months, the first time they received non-nutritive liquids (water, tea, juice, or both), formula milk, and/or solid foods. From these data, we constructed the outcome variable “breastfeeding practices,” composed of four categories: (1) exclusive breastfeeding (EBF, breastfeeding with no other food or drink, not even water); (2) predominant breastfeeding (mainly breast milk but with other liquids, such as water and water-based drinks or fruit juice); (3) mixed feeding (formula milk, liquids and/or solid foods in addition to breast milk); and (4) no breastfeeding (having stopped breastfeeding completely). Constructing the outcome variable in this way allowed us to describe not merely current breastfeeding practices but how they changed over the 1st months of infants' lives.

Then, to explore which factors condition EBF, we first classified participants in two groups according to the duration of breastfeeding: the early abandonment of exclusive breastfeeding group ( ≤ 1 m-EBF) included women who practiced EBF for <1 month, while the beyond 1 month EBF group (>1 m-EBF) included those who practiced EBF for more than 1 month. We selected 1-month as a cut off point for creating groups because very early EBF desertion was common in this population. We then compared women in the ≤ 1 m-EBF -EBF with those in the >1 m-EBF in terms of two groups of factors: (1) sociodemographic characteristics, (2) maternal and infant factors.

#### Sociodemographic Characteristics

These factors included: participant's age (years), whether she lived with the baby's father (yes/no), if it was important for the baby's father that she breastfed (agree/disagree), occupation (housewife/work outside home), household type (monoparental/nuclear/extended), schooling (years), whether she had governmental social security (Seguro Popular/IMSS/ISSSTE) or was beneficiary of any government support program (yes/no), household welfare level and household food security.

We estimated household welfare levels using the AMAI rule 8X7, a tool developed by the AMAI (in Spanish, Mexican Association of Market Intelligence and Public Opinion Agencies). It consists of eight items and classifies households in seven socioeconomic levels according to the head of the household's ability to satisfy their members' needs (11). Since we observed that women in the higher levels tended to abandon EBF earlier, we collapsed the seven resulting levels into two broad categories according to income distribution: a higher, privileged segment which dedicates a greater proportion of their spending to education, entertainment, communication, saving and automobile acquisition (scores A/B, C+, C, and C-), and a lower, underprivileged group which spends mainly on food and drinks, transport and personal care (scores D+, D, and E) (11).

We evaluated food security using the Latin American and Caribbean Scale of Food Security (ELCSA), which consists of 15 questions with “yes” or “no” answers. It classifies households in four categories (food security, mild food insecurity, moderate food insecurity and severe food insecurity) according to the women's opinion and experience regarding their difficulty to access food as a result of lack of money or other resources.

#### Maternal/Infant Factors

Maternal and infant factors comprised a wide array of variables related to women's reproductive history, last pregnancy, birth and hospitalization; and also early postpartum factors, breastfeeding experiences and beliefs.

Regarding the participant's reproductive history, we asked the number of previous liveborn children. With regard to the participant's previous child, factors included: age of the previous child at the time of the study (years), length of exclusively breastfeeding her previous child (months), and whether she had been satisfied with her previous experience (yes/no).

Participant's last pregnancy factors referred to the baby they were currently breastfeeding, and included: whether it was a planned pregnancy (yes/no), the moment of her first prenatal care visit to FLHU (gestational weeks), number of prenatal care visits to FLHU, received information about EBF until 6 months old (yes/no), developed gestational diabetes or hypertensive disorder of pregnancy (yes/no).

Birth factors included: place of delivery (third level hospital, other public hospitals, private clinic, home), mode of delivery (vaginal/cesarean section), whether the birth was attended by medical staff (yes/no), hospitalization length (hours), baby's sex, gestational age at birth (weeks), if the baby was premature (yes/no), weight at birth (kg), and length at birth (cm).

Hospitalization factors included: initiated breastfeeding within the 1st hour (yes/no), roomed-in with baby (yes/no), problems with breastfeeding during hospitalization (yes/no, cause), offered liquid other than breast milk during hospitalization (yes/no), and used pacifier or bottle nipple during hospitalization (yes/no).

Early postpartum factors included: number of visits to FLHU for infant follow-up, received breastfeeding information/support during postpartum visits to FLHU (yes/no).

Breastfeeding factors included: duration of exclusive breastfeeding (weeks), reasons to stop exclusive breastfeeding. Additionally, we explored participants' thoughts and beliefs about breastfeeding and formula milk. We asked participants if they agreed with the following statements (yes/no): “*I am convinced that giving only breast milk until the baby is 6 months old, without giving any other food, is the best for her/him,” “When you finish breastfeeding, you are always sure that your baby under 6 months is full,” “Formula milk is an important food to accompany breast milk before 6 months,” “When the baby is not full, you should give her/him powdered milk or some other food, even if she/he is*<*6 months old.”*

## Data Analysis

We performed exploratory data analysis in all variables as well as normality tests in continuous variables to analyze whether they had normal distributions. For evaluating selection bias, we compared sociodemographic, pregnancy, birth and hospitalization variables from included and not included women using Student's *t*-test or Mann-Whitney *U*-test depending on variable distribution.

For the statistical analyses, first, we performed a bivariate analysis to establish the association between the outcome variables and each independent variable. We used Chi squared, Student's-*t* or Mann-Whitneys's *U*-test depending on the type and distribution of the potentially influencing factor. Independent variables that were associated with the outcome variable (*p* ≤ 0.10) were included in backward stepwise logistic regression models. We checked the uptake of variables for collinearity and accepted correlations > 0.35, tolerance > 0.79 and variance inflation factor (VIF) <1.27.

In order to explore which factors were associated with the duration of EBF, we conducted two logistic regression models; in both of them the predicted probability was for being in the >1 m-EBF group.

In model 1 we explored sociodemographic factors, which included: lives with the baby's father, occupation, and household food security. In model 2 we included maternal / infant factors that showed association with the outcome variable (>1 m-EBF) in the bivariate analyses. In this model we included sociodemographic factors as confounding variables in order to minimize or eliminate possible residual confounding.

Finally, to propose a conceptual model that describes the association of studied factors with EBF, we performed a bivariate analysis among the independent factors, using Chi squared, Student's-*t* or Mann-Whitney's *U*-test depending on variable type and distribution.

## Results

In this study, we aimed (1) to describe breastfeeding practices among women from semi-rural communities in southeast Mexico; and (2) to explore which factors, modifiable or not, condition such practices.

### Study Sample

We invited a total of 200 women to participate, 57 (28.5%) of which did not meet the inclusion criteria: 24 had not received their prenatal care at CESSA, 22 had babies older than 6 months, eight had had complications during birth and three did not accept to participate. Therefore, 143 women were included in the final sample.

We compared our final sample to the group of women not included in the study. There were no differences in relation to most sociodemographic characteristics evaluated, except that more included women had the type of governmental social security whose beneficiaries are people without formal employment (Seguro Popular) (97.9%, *n* = 140 vs. 87.7%, *n* = 50; *p* < 0.01). A higher proportion of women in the not included group were beneficiaries of IMSS/ISSSTE, governmental social security for people with formal employment. This difference was anticipated since the latter women were expected to have their prenatal care in their designated clinics, unlike those with Seguro Popular who would be attended at CESSA or FLHU units.

A higher proportion of not included women experienced complications during pregnancy (31.6%, *n* = 18 vs. 15.4%, *n* = 22; *p* = 0.01); gave birth through C-section (43.9%, *n* = 25 vs. 21.0%, *n* = 30; *p* < 0.01) and were hospitalized after giving birth (28.1%, *n* = 16 vs. 8.5%, *n* = 12; *p* < 0.01). Hospital stay in hours was different (median 24, p25–p75 24–75 vs. 24, 17–39; *p* = 0.01). These differences also reflect selection criteria since we did not include women who have had conditions that could be a barrier for breastfeeding initiation. Women with such barriers would be expected to have gestational complications, deliver by cesarean section and/or had longer hospital stay.

### Breastfeeding Practices

**Figure 2:**
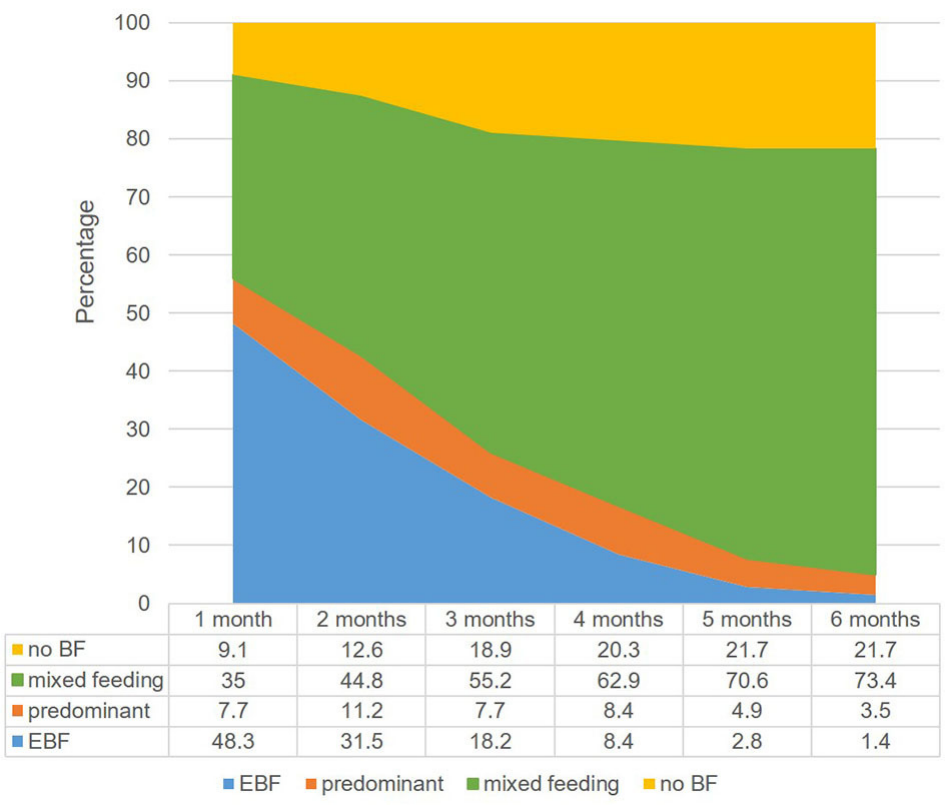
Breastfeeding practice trends. Numbers in the figure's table represent percentages.

Figure 2 shows that by the end of the 1st month of life, half of the women (51.7%, *n* = 74) in the study had abandoned EBF, some (7.7%, *n* = 11) had introduced non-nutritive liquids; most (35%, *n* = 50) had introduced milk formula or other food (9.1%, *n* = 13) and others had stopped breastfeeding completely. As months went by, EBF practice fell sharply and mixed feeding grew importantly.

Since EBF was abandoned by an important proportion of women as early as the 1st month of life, we wanted to find out which factors might be associated with this early abandonment.

### Sociodemographic Characteristics

Table 1 shows the sociodemographic characteristics of the study population, comparing the ≤ 1 m-EBF and >1 m-EBF groups. Significantly more women in the >1 m-EBF were living with their baby's father, were housewives, and lived in households in the lower welfare level or with some level of household food insecurity.

**Table 1:** Sociodemographic characteristics.

	**Total**	**≤1 m-EBF (*n* = 63)**	**>1 m-EBF (*n* = 80)**	***p***
**Age**				
Years	23.0 (19.0 - 27.0)	22.0 (19.0 - 27.0)	23.0 (20.0 - 27.7)	0.45
**Lives with the baby's father**				
Yes	123 (86%)	49 (77.8%)	74 (92.5%)	**0.01**
**It is important for the baby's father that you breastfeed**				
Agree	123 (86%)	50 (79.4%)	73 (91.3%)	**0.05**
**Occupation**				
Housewife (vs. work outside home)	127 (88.8%)	52 (82.0%)	75 (93.8%)	**0.03**
**Household type**				
Monoparental	4 (2.8%)	3 (4.8%)	1 (1.3%)	0.10
Nuclear	79 (55.2%)	29 (46%)	50 (62.5%)	
Extended	60 (42%)	31 (49.2%)	29 (36.3%)	
**Schooling**				
Years	9.0 (8.0 - 12.0)	9.0 (8.0 - 12.0)	9.0 (8.2 - 12.0)	0.68
**Governmental Social Security**				
Yes	140 (97.9%)	62 (98.4%)	78 (97.6%)	0.74
**Household welfare level**				
Lower level	105 (73.4%)	41 (65.1%)	64 (80.0%)	**0.04**
**Household food security**				
Secure	33 (23.1%)	19 (31.1%)	14 (17.1%)	**0.04**
Mild insecurity	68 (47.6%)	29 (46.0%)	39 (48.8%)	
Moderate insecurity	26 (18.2%)	11 (17.5%)	15 (18.8%)	
Severe insecurity	16 (11.2%)	3 (4.8%)	13 (16.3%)	

*Data shows number of cases (%) or median (p25–p75). We compared continuous variables between groups using Student's t-test or Mann-Whitney's U-test, and categorical variables using Chi squared test*.

*Bold values indicate statistically significant differences (p <0.05) between study groups*.

As expected, there was an important correlation between the household welfare and level and food security (*r* = −0.36). More women classified in the high level of household welfare lived in a food secure household (44.7%, *n* = 17 vs. 15.2%, *n* = 16); there was a similar proportion of mildly insecure households between the two categories of welfare (47.7%, *n* = 18 vs. 47.6%, *n* = 50) and a lower proportion of moderately (7.9%, *n* = 3 vs. 21.9%, *n* = 23) and severely insecure (0%, *n* = 0 vs. 15.2%, *n* = 16) (*p* < 0.01). Another correlation was present between the variables “Lives with the baby's father” and “*It is important for the baby's father that you breastfeed*” (*r* = −0.42, *p* < 0.01), as more women who lived with the baby's father said he was interested in BF (91.9%, *n* = 113 vs. 50%, *n* = 10; *p* < 0.01); therefore, we did not include the variables household welfare level and “*It is important for the baby's father that you breastfeed*” in the following logistic regression analysis.

In a model adjusted by the significantly different variables in bivariate analysis, women living with the baby's father and with severe household food insecurity, were more likely to breastfeed beyond the 1st month (model 1 in **Table 4**).

**Figure 3:**
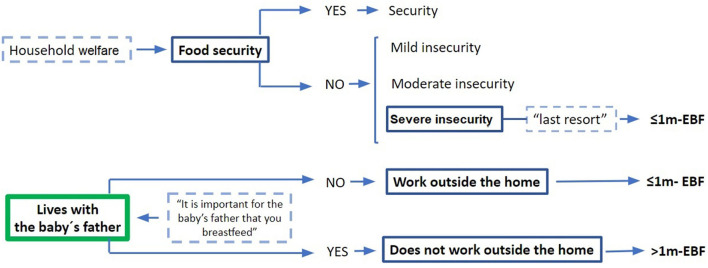
Conceptual model of socioeconomic factors related to EBF.

In order to propose a conceptual model about how sociodemographic characteristics influence EBF, we further analyzed the possible associations between them (Figure 3). For example: only 10% (*n* = 10) of women living with the baby's father worked outside home, compared to 30% (*n* = 6) of those not living with him (*p* = 0.01) (*r* = −0.24, *p* = 0.01). There was no difference in the proportion of the women who worked outside their home or lived with the baby's father between the food security categories.

### Maternal/Infant Factors

In Table 2 we show information about the women's reproductive history and last pregnancy, including previous breastfeeding experience. Significantly more women in the >1 m-EBF had at least one previous liveborn baby. Also, more women in this group were diagnosed with GDM or a hypertensive disorder of pregnancy. In contrast, more women in the ≤ 1 m-EBF had never breastfeed or had stopped EBF before 1 month with their previous child, delivered by a cesarean section and had a higher BMI at the time of the study visit.

**Table 2:** Reproductive history and last pregnancy factors.

	**Total**	**≤1 m-EBF (*n* = 63)**	**>1 m-EBF (*n* = 80)**	***p***
**Reproductive history**				
Has at least one previous liveborn baby	86 (60.1%)	31 (49.2%)	55 (68.8%)	**0.02**
**Previous baby**				
Any breastfeeding	72 (83.7%)	24 (77.4%)	48 (87.3%)	0.23
No previous BF or early EBF termination	98 (68.5%)	51 (81.0%)	47 (58.8%)	**<** **0.01**
Overall BF duration (months)	8.50 (1.75 - 15.5)	7.0 (1.0 - 14.0)	12 (4.0 - 18.0)	0.19
Satisfied with BF experience (yes)	22 (25.6%)	9 (29.0%)	13 (23.6%)	0.58
**Current pregnancy**				
Planned pregnancy (yes)	65 (45.5%)	30 (47.6%)	35 (43.8%)	0.64
First prenatal care visit before 8 wk gestation	69 (48.3%)	27 (42.9%)	42 (52.5%)	0.25
At least five prenatal care visits	111 (77.6%)	47 (74.6%)	64 (80.0%)	0.44
Received information about EBF until 6 mo	65 (45.5%)	32 (50.8%)	33 (41.3%)	0.25
Pregnancy complications (GDM, HDP)	22 (15.4%)	5 (7.9%)	17 (21.3%)	**0.03**
Delivery mode (cesarean section)	30 (21%)	19 (31.1%)	11 (13.4%)	**0.01**
Maternal BMI	26.42 ± 5.49	27.61± 6.03	25.49 ± 4.88	**0.02**

*GDM, gestational diabetes mellitus; HDP, hypertensive disorder of pregnancy. Data shows number of cases (%), mean ± s.d. or median (p25–p75). We compared continuous variables between groups using Student's t-test or Mann-Whitney's U-test, and categorical variables using Chi squared test*.

*Bold values indicate statistically significant differences (p <0.05) between study groups*.

Regarding prenatal care, most women attended at least five prenatal visits to the health care service during pregnancy, around half of them went for the first time during the first 8 weeks of gestation. During these visits 59.4% (*n* = 85) received information about the importance and benefits of breastfeeding; 45.4% (*n* = 65) were counseled to exclusively breastfeed till their baby was 6 months; 8.4% (*n* = 12) were told to practice on-demand breastfeeding and 45.4% (*n* = 65) were taught how to position the infant on the breast. Neither prenatal care attendance nor the various types of received information were statistically different between the ≤ 1 m-EBF and >1 m-EBF groups.

None of the participants reported smoking and only 2.1% (*n* = 3) drank alcohol at the time of the study visit.

Regarding pregnancy resolution, most women (85.3%, *n* = 122) gave birth at the High Speciality Regional Hospital for Women in Villahermosa City; a minority gave birth at another public hospital (4.9%, *n* = 7), or a hospital ran by the Mexican Institute of Social Security (5.6%, *n* = 8), or a private clinic (2.1%, *n* = 3) or at home (2.1%, *n* = 3). Likewise, most births were attended by a health professional (97.9%, *n* = 140), and required short hospital stays (median 24 h, p25–p75 17.50–39.00). There was no difference between ≤ 1 m-EBF and >1 m-EBF groups regarding birthplace (*p* = 0.58), birth attendants (*p* = 0.42), and hospitalization length (*p* = 0.32).

Table 3 shows the comparison of newborn characteristics between ≤ 1 m-EBF and >1 m-EBF groups. Regarding infant sex, gestational age, prematurity, weight and length at birth, only gestational age at birth was different between study groups.

**Table 3:** Newborn characteristics.

	**Total**	**≤1 m-EBF (*n* = 63)**	**>1 m-EBF (*n* = 80)**	***p***
Girls (*n* = 143)	76 (53.1%)	34 (54.0%)	42 (52.5%)	0.86
Gestational age (weeks) (*n* = 140)	40 (38 - 42)	41 (39 - 42)	40 (38 - 42)	**0.03**
Premature (*n* = 140)	5 (3.6%)	1 (1.6%)	4 (5.1%)	0.38
Weight at birth (kg) (*n* = 140)	3.10 (2.81 - 3.50)	3.12 (2.90 - 3.47)	3.10 (2.80 - 3.50)	0.52
Length at birth (cm) (*n* = 80)	50.0 (48.0 - 52.0)	50.5 (49.0 - 52.0)	49.0 (48.0 - 51.0)	0.07

*Some answers were not included because they were not plausible or the woman did not remember. Data shows number of cases (%) or median (p25–p75). We compared continuous variables between groups using Student's t-test or Mann-Whitney's U-test, and categorical variables using Chi squared test*.

*Bold values indicate statistically significant differences (p <0.05) between study groups*.

**Figure 4:**
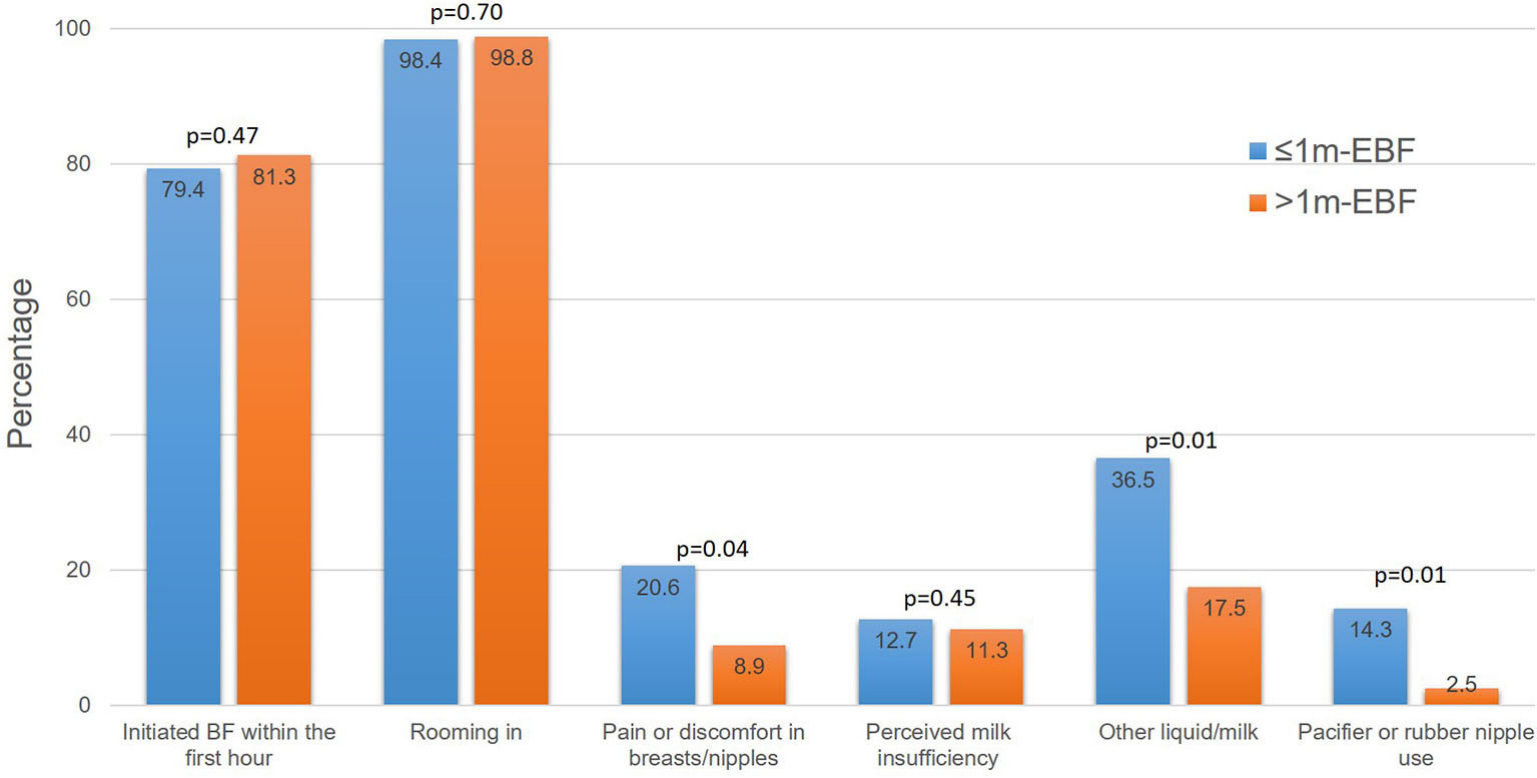
Breastfeeding practices during hospitalization. Groups were compared using the chi-square test.

Figure 4 shows breastfeeding related factors during participants' hospitalization. Most women initiated breastfeeding within the 1st hour after giving birth, were roomed in with their babies and perceived sufficient milk production; there was no difference between groups on these variables. In contrast, significantly more women in the ≤ 1 m-EBF group experienced pain or discomfort on their breasts and/or nipples, gave their newborn a liquid food other than her own milk and used a nipple or pacifier during hospital stay.

There was an important correlation between the variable “gave liquid other than her own milk” and variables “used a nipple or pacifier” (*r* = −0.49, *p* < 0.01) and “had pain or discomfort in breasts/nipples” (*r* = 0.35, *p* < 0.01); therefore, we did not include the last two variables in the logistic regression model. Also, the variable length-at-birth was not included due to the large number of missing data.

Between birth and the study visit, more women in the >1 m-EBF had attended the CESSA or FLHU at least three times (87.5%, *n* = 70 vs. 76.2%, *n* = 48; *p* = 0.07), primarily to receive vaccination for their baby (95.8%, *n* = 137). Around a fifth of the women in our sample recalled receiving breastfeeding information/support during their postpartum visits to the health facilities ( ≤ 1 m-EBF 17.5%, *n* = 11 vs. >1 m-EBF 21.2%, *n* = 17; no difference between groups). When the women who received information, was asked about the type of information they had received, more women in the >1 m-EBF (53.5%, *n* = 15 vs. 21.4%, *n* = 6; *p* = 0.06) recalled correct information such as the importance of EBF until the baby turned 6 months or breast massages to alleviate breast discomfort. However, women also remembered erroneous information such as to offer formula milk if the baby remained hungry after a feed, or to give clean drinking water if the baby was thirsty.

More than half of our study population (61.5%, *n* = 88) also attended other health facilities besides CESSA or FLHU; most of them went to a private physician (45.5%, *n* = 40) or a pharmacy (52.3%, *n* = 46), mainly because of child's sickness. At these alternative facilities, 23.1% of participants (*n* = 33) received information about breastfeeding; unfortunately, we didn't ask what kind of information they received. Study groups were not different considering postpartum visits to health facilities or received information.

**Figure 5:**
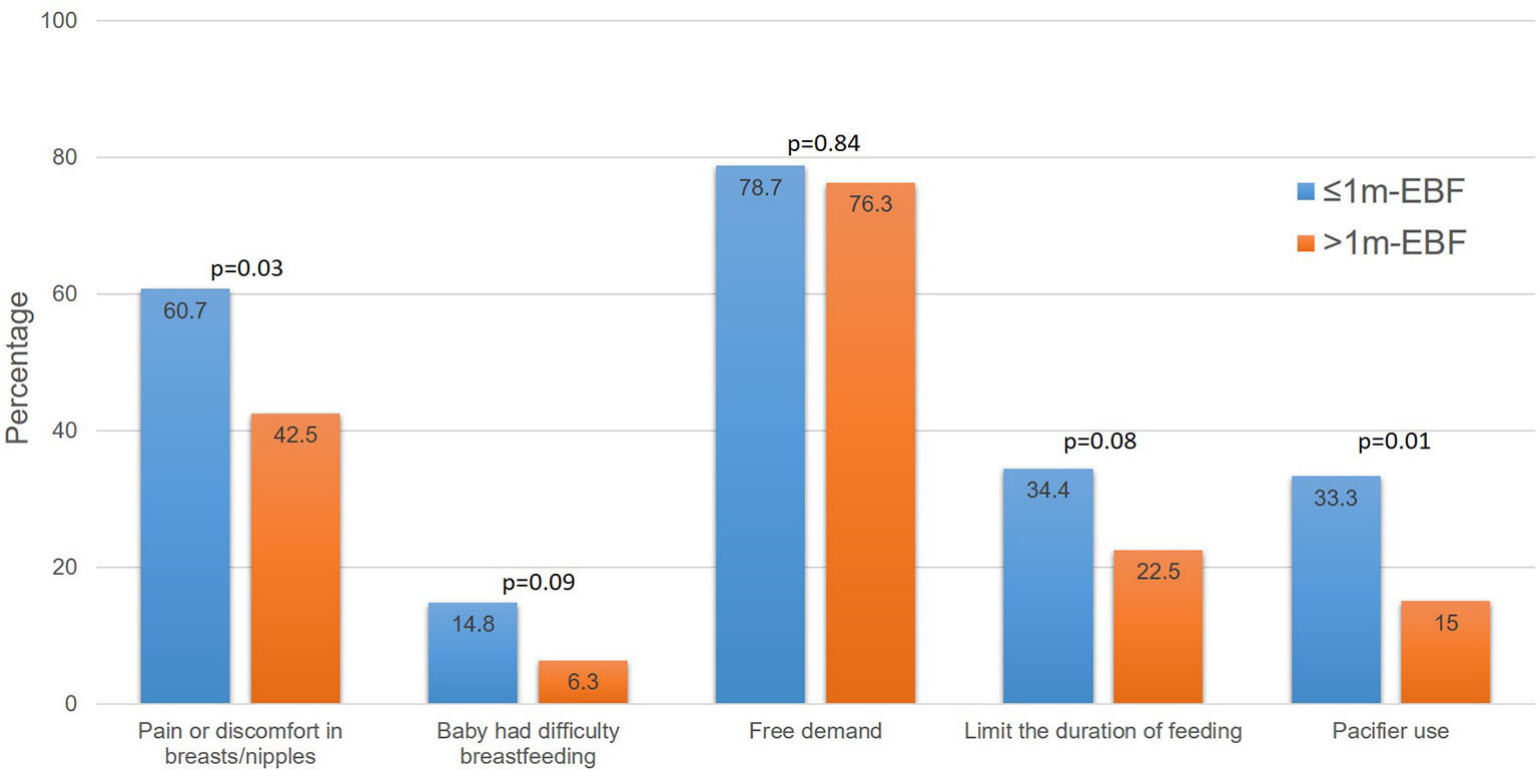
Breastfeeding during early postpartum. Groups were compared using the chi-square test.

Figure 5 shows breastfeeding practices after leaving the hospital. Most of the study participants practiced on demand breastfeeding. More women in the ≤ 1 m-EBF group limited the time they let their infant suck at their breast, reported their baby had difficulties to latch and breastfeed correctly, experienced breast and/or nipple pain, and gave a pacifier to their infants.

More women in the L-EB group stopped breastfeeding due to an illness (18.8%, *n* = 15 vs. 7.9%, *n* = 5; *p* = 0.06). The ailments they presented were common infections, like cold, vaginal or tooth infections (6.3%, *n* = 9); mosquito transmitted viral diseases such as dengue or chikungunya (4.2%, *n* = 6), herpes zoster (not on the mammary gland, 0.7%, *n* = 1), anemia (0.7%, *n* = 1) or colitis (0.7%, *n* = 1). None of these illnesses contraindicated breastfeeding. It is interesting to note that most of the participants who stopped, did resume breastfeeding upon recovery (15%, *n* = 12 vs. 3.8%, *n* = 3; *p* < 0.01).

Regarding the participants' thoughts about formula milk and breastfeeding, more women in the ≤ 1 m-EBF group agreed with the phrases “*Formula milk is an important food to accompany breast milk before 6 months*” (66.7%, *n* = 42 vs. 37.5%, *n* = 30; *p* < 0.01) and “*When the baby is not full, you should give her/him powdered milk or some other food, even if she/he is*<*6 months old*” (66.7%, *n* = 42 vs. 42.5%, *n* = 34; *p* = 0.02). There was no difference between groups in the proportion of women who agreed with the phrases “*When you finish breastfeeding, you are always sure that your baby under 6 months is full*” (58.7%, *n* = 37 vs. 70.0%, *n* = 56; *p* = 0.37) and “*I am convinced that giving only breast milk until the baby is 6 months old, without giving any other food, is the best for her/him*” (60.3%, *n* = 38 vs. 72.5%, *n* = 58; *p* = 0.30).

There was an important correlation between the variable “*When the baby is not full, you should give her/him powdered milk or some other food, even if she/he is*<*6 months old*” and variables “*Formula milk is an important food to accompany breast milk before 6 months*” (*r* = 0.413, *p* < 0.01). Therefore, we did not include the last variable in the second logistic regression model.

To explore the interaction of those factors most strongly associated with >1 m-EBF, we performed a second logistic regression model including maternal/infant factors that resulted statistically significant in the bivariate analyses and adjusted for sociodemographic variables as confounders (model 2 in Table 4). When such interaction was considered, household food security was no longer significantly associated with >1 m-EBF. Overall, women who lived with the baby's father, had complications during pregnancy, delivered vaginally or attended a health center at least three times postpartum were more likely to practice >1 m-EBF. Conversely, women with larger bodies (i.e., higher BMI); who gave other liquids during their hospital stay; who had pain in their breasts or used a pacifier after hospitalization; and who believed that you should give the infant powdered milk or some other food when the baby is not full, were less likely to practice EBF beyond 1 month. Having no previous BF experience and limiting the duration of the feed marginally reduced the likelihood of >1 m-EBF.

**Table 4:** Logistic regression models for factors associated with L-EBF.

	**Model 1**		**Model 2**	
	**Sociodemographic characteristics**		**Global model**	
	**OR (95% CI)**	***p***	**OR (95% CI)**	***p***
**Household food security**				
Secure	1	**0.03**		
Marginally insecure	2.08 (0.88 - 4.96)	0.09		
Moderately insecure	2.45 (0.82 - 7.30)	0.10		
Severely insecure	9.93 (2.09 - 47.26)	**0.01**		
Lived with the baby's father (yes)	4.93 (1.58, 15.37)	**<0.01**	3.83 (1.09, 13.37)	**0.03**
GDM or HDP (yes)			6.32 (1.41, 28.27)	**0.02**
No previous BF experience or EBF <1 mo			0.35 (0.12, 1.02)	0.05
Maternal BMI at the time of study			0.91 (0.84, 0.98)	**0.02**
Received other liquid in the hospital (yes)			0.32 (0.11, 0.92)	**0.03**
Vaginal delivery			3.21 (1.03, 9.93)	**0.04**
Attended health center at least three times postpartum (yes)			3.24 (1.06, 9.89)	**0.04**
Had pain or discomfort in breasts/nipples after hospital discharge (yes)			0.31 (0.12, 0.80)	**0.01**
Limits the duration of the feed (yes)			0.37 (0.13, 1.01)	0.05
Pacifier use after hospital discharge			0.31 (0.10, 0.94)	**0.04**
“*When the baby is not full, you should give her/him powdered milk or some other food, even if she/he is <6 months old”* (agree)			0.22 (0.0.08, 0.55)	**<0.01**

*Model 1. Sociodemographic characteristics: variables not included in the model: occupation (stay-at-home mother or work away from home)*.

*Model 2. Global model variables not included in the final model: household food security, occupation, gestational age and baby had difficulty breastfeeding*.

*In both models, the predicted probability is for being in the L-EBF group*.

*Bold values indicate statistically significant differences (p <0.05) between study groups*.

**Figure 6:**
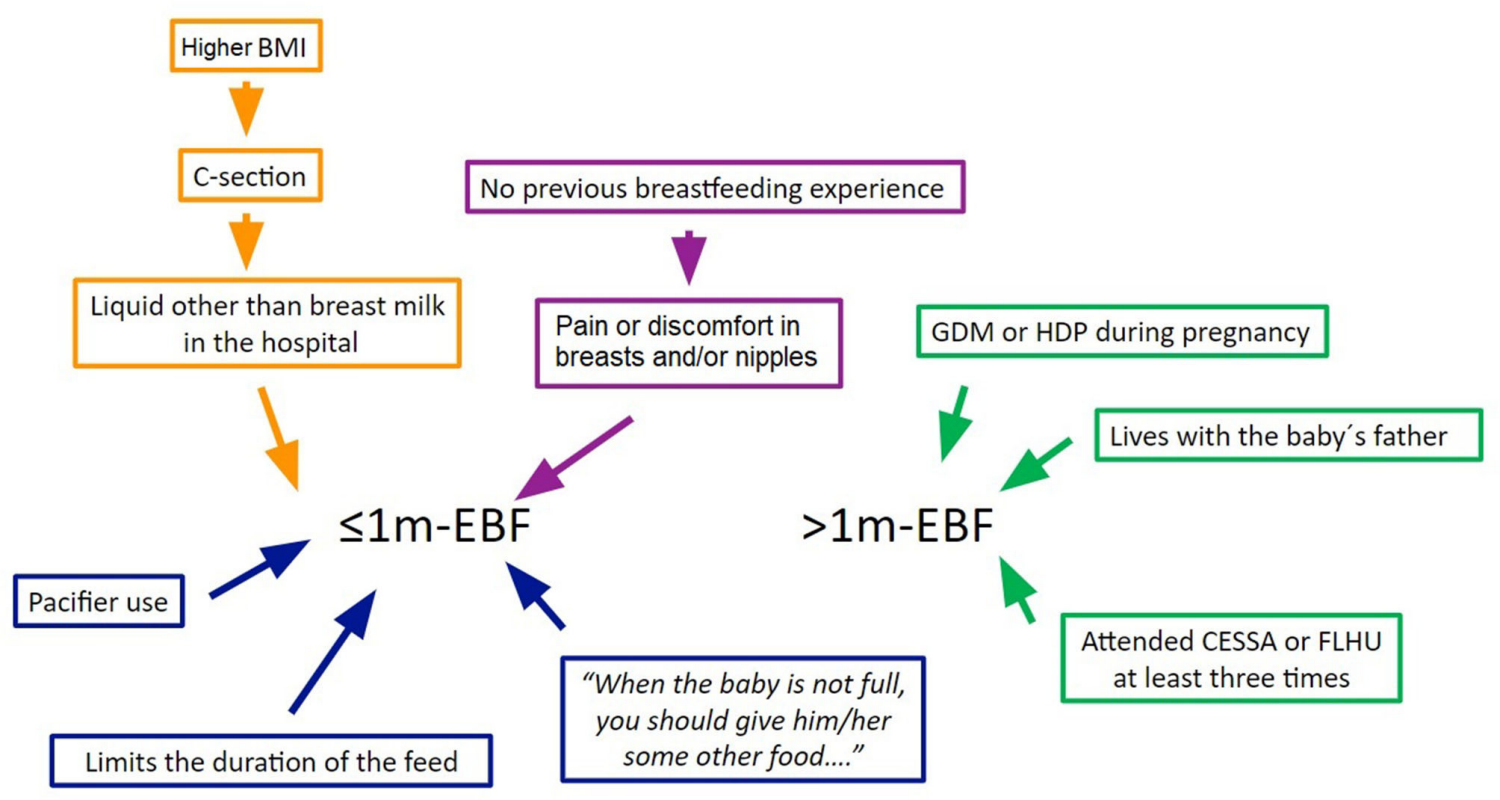
Conceptual model of maternal / infant factors related to EBF. ≤ 1 m-EBF = up to 1 month exclusive breastfeeding; >1 m-EBF = beyond 1 month exclusive breastfeeding; GDM, gestational diabetes mellitus; HDP, hypertensive disorder of pregnancy.

Lastly, in order to illustrate how the variables in our global model may influence >1 m-EBF, and propose a conceptual model, we analyzed the association between them (Figure 6). We found that less than half (20.4%, *n* = 23) of the infants delivered vaginally received a liquid different to breast milk as opposed to infants delivered by C-section (46.7%, *n* = 14) (*p* = <0.001) (*r* = 0.24, *p* < 0.01). Compared to women with no previous BF experience (59.4%, *n* = 57), fewer women who had breastfed before (31.1%, *n* = 14) suffered pain or discomfort in breasts and nipples (*p* < 0.01) (*r* = −0.26, *p* < 0.01). More women who agreed that “When the baby is not full, you should give … other food …” gave her infant a pacifier (30.3%, *n* = 23) as opposed to those who did not agree (14.9%, *n* = 10) (*p* = 0.02) (*r* = −0.18, *p* = 0.04). Women who delivered by C-section had higher BMI than those delivering vaginally (28.57 ± 5.34 vs. 25.85 ± 5.42, *p* = 0.01). Similarly, women that gave their infants liquids different to breast milk during hospitalization had higher BMI than those who did not (28.58 ± 6.39 vs. 25.67 ± 4.96, *p* < 0.01). Finally, women with no previous BF experience had lower BMI (25.15 ± 6.14 vs. 27.26 ± 4.88, *p* = 0.02); however this association was not taken into account in the conceptual model since many women in the inexperienced group were primiparas and parity is associated with weight retention and increased BMI (12).

## Discussion

The results of our work show that very early abandonment of EBF during the 1st month postpartum is a common practice in the community; by the end of the 1st month, half of the women had stopped practicing it. A previous study held in the Mexican states of Puebla and Chihuahua, using the WHO status quo EBF indicator, also documented that by the end of the 1st month, 55.2% of women had stopped EBF their infants (13). However, this trend may not be clearly appreciated from the national data obtained in surveys, since the gross indicator of “exclusive breastfeeding under 6 months” which is suggested by the World health Organization (WHO) and UNICEF aggregates all infants 0–5 months to calculate the percentage who were exclusively breastfed the day previous to the survey.

It is very relevant that such a large proportion of women in these communities decide to stop EBF from such an early stage, so our findings contribute to understanding which factors are associated with this inadequate practice in order to design and target breastfeeding interventions at the community and individual levels.

In our study there were four sociodemographic factors associated with exclusive breastfeeding beyond the 1st month of life: the mother “living with the infant's father,” living in a household with low welfare level or with food insecurity, and the mother staying at home (instead of going out to work). However, when the first logistic regression was performed, occupation was dismissed from the model, “living with the baby's father” and living in a severely insecure household increased the odds to breastfeed beyond the 1st month. In the global logistic regression model, the only sociodemographic variable that explained EBF beyond 1 month was “living with the baby's father.” The conceptual model (Figure 3) suggests pathways in which these factors influence EBF duration.

Regarding the association between EBF duration and household food insecurity, it has been documented that households with the lowest welfare and incomes, which also tend to have members with the lowest rates of education and access to goods and services, are often more likely to be food insecure (14–16). These households would particularly benefit from a longer duration of EBF in two ways: firstly, because of its protective role on the nutritional, physical and emotional health of both infant and mother; secondly, to avoid expenses that the family would have to make to face an illness: visits to the health services, medicines, as well as the indirect costs caused by the absenteeism of the parents to care for the sick child. It would also protect the household's economy by avoiding the purchase of breast milk substitutes (15, 17, 18).

In our study, severe food insecurity was an independent predictor of EBF practice beyond the 1st month of life, even when other sociodemographic factors were taken into account. The “positive” effect of food insecurity on breastfeeding indicators, such as BF initiation and duration, has been observed before (19, 20). In particular, the influence of extreme food insecurity on EBF was documented through qualitative data in a study held in Haiti (21, 22). Some mothers continued BF when they could not afford to buy other foods for their infants, i.e., formula milk or other complementary foods. These women continued with EBF not by decision but as a “last resort.” forced by not having money to give them something else.

However, when food insecurity was not severe, we found no association with EBF beyond the 1st month postpartum. This may be the result of the coincidence of positive and negative effects of food insecurity on EBF that nullified any observation. Previous studies have documented some beliefs associated with food insecurity that may stimulate a woman's decision to stop EBF or any type of BF. For example, when there is not enough food for the woman to eat, her concern is that her milk would be insufficient and of low quality (with not enough nutrients) to adequately nourish her infant (21, 23). Another concern is that negative emotions induced by food insecurity such as stress, could either pass to the infant during breastfeeding, and affect his/her appetite or mood; or directly reduce the woman's milk production, becoming insufficient for her baby (23).

Regarding maternal occupation, work outside home has been linked to the reduction of EBF (21, 24, 25). In Mexico, women working in the formal sector have paid maternity leave for 12 weeks in total, divided in two 6 weeks periods, before and after birth. And with prior authorization, up to 4 of the 6 weeks off before delivery can be transferred to the postpartum period. This means that they can spend between 1.5 and 2.5 months postpartum at home (26); however this time is still not long enough to protect breastfeeding (3). Women working in the informal sector, that is, women who are self-employed or have a non-salary contractual arrangement, are not entitled to a paid maternity leave (27). Most women in our study belonged to this informal economic sector (93.7%) and therefore lacked income security during lactation. These women belong to the most socioeconomically disadvantaged sectors, and possibly face the need to return to work early (21). Concomitantly, women in these circumstances may experience unfavorable work conditions that have been found to interfere with EBF breastfeeding. For example, lack of job flexibility, long commute time to work, lack of support, no guaranteed scheduled breaks or a suitable facility to breastfeed or extract and store milk (15, 28, 29).

In our study, we found that living with the infant's father increased the probability of EBF beyond the 1st month of life. This positive influence of a partner or spouse to BF practices has also been observed in previous studies (13, 30). The role of the father might be related to the practice of EBF, in at least in two ways: first, the woman's perception about her partner's attitudes and beliefs about BF may influence her own attitudes and decisions about their child's feeding. Second, by the emotional and instrumental support he provides to the BF woman. For example, giving praise and encouraging compliments and by making the mother feel comfortable and giving practical assistance in household chores (31, 32). Although we did not look at the kind of support the fathers might have given to the women in our study, one interesting observation was that “living with the baby's father” was the only significant sociodemographic variable in the global model. This suggests that the father's presence in the household may be protective of the more severe form of food insecurity and possibly reduce, or at least postpone, the woman's need to go back to work (33), in turn having a positive effect on EBF.

We were able to identify several maternal and infant factors associated with the duration of EBF, from non-modifiable pregnancy and hospitalization conditions beyond women's control, to modifiable practices and beliefs. Based on the associations between these factors, we were able to propose a conceptual model to describe their interactions (Figure 6).

In our study population, women whose last pregnancy was complicated by GDM or a hypertensive disorder were more likely to practice EBF beyond 1 month. This was an unexpected result since other studies have shown that women with GDM are less likely to practice EBF or, if they do, it is usually for shorter periods than other mothers (34).

However, this result could suggest that, due to their condition, those women may have received special information, either during their prenatal care or postpartum visits, on the benefits of EBF on diabetes and hypertension. Indeed, BF has been shown to improve the glycemic status of women with previous GDM through lowered rates of impaired glucose tolerance and lower fasting plasma glucose (35). Such women have a lower risk of developing type 2 DM than those who had GDM but did not breastfeed (36, 37). Similarly, BF is associated with a lower risk of maternal hypertension, especially when practiced for more than 1 month (38), and exerts a protective effect against migraine attacks, a condition closely related to hypertensive disorders (39).

Although we asked participants whether they received information during their prenatal care about the benefits of BF, we did not delve into the details of such information and therefore cannot know if EBF was mentioned as a protective factor against DM or hypertensive disorders. However, since Mexican law requires health professionals to closely monitor women with such complications during and after pregnancy (40), it is likely that participants may have received related information during their prenatal care.

Regarding the mothers' previous experience with EBF, this variable was significantly associated with EBF beyond 1 month. Maternal previous experience has also been shown to influence both the intention and the actual practice of BF in other populations (41–45). However, in our study, maternal previous experience was marginally associated with >1 m-EBF when the interaction of other early postpartum and breastfeeding factors was taken into account in the logistic regression models. Our results in this regard may suggest that while previous experience may predispose the mother toward a positive intention to adequately BF, it may be outweighed by the challenges she endures in her current BF practice; we will discuss this further ahead.

It is interesting to notice that the information about EBF that women received during their prenatal care was not associated with the duration of EBF. Considering this in conjunction with the fact that living with the baby's father and his interest in BF were indeed related with >1 m-EBF, it may seem that the attitudes and information provided by the mother's close social group may be more influential in her BF practice than what health professionals may tell her. In this regard, Humphreys et al. reported that low-income women in the southwest USA were less influenced in their infant feeding decisions by health professionals' attitudes than by the attitudes and beliefs of members of their social support networks, including family members, the baby's father, and lactation consultants (44). Further studies about the structure and dynamics of the social networks in particular contexts may provide new insights into the women's influences and beliefs, as well as allowing for the design of interventions that target not only the mother but also key members of her social group.

Women whose infants received liquids other than breastmilk during their hospital stay were also less likely to practice >1 m-EBF. While studies usually focus on the introduction of liquids during the 1st months of infants' lives, few report on this practice during the neonatal hospital stay. The only other study we have found reporting this practice was carried out in Istanbul, Turkey, where researchers found a similar negative association between introducing formula milk during the hospital stay and duration of EBF (46).

Providing neonates with liquids other than their mother's breast milk during hospital stay, including formula milk or breastmilk from a milk bank, is only indicated when either infant or mother courses through a medical condition that limits their capacity to breastfeed. These include infant metabolic diseases, extreme prematurity, maternal HIV/hepatitis/herpes infections or substance abuse, or undergoing an emergency medical procedure (47). Nevertheless, a quarter of the infants in our study were given liquids other than breastmilk during their newborn hospital stay, although none of the participants or their babies had any condition that limited BF and maternal/infant hospitalization was a non-inclusion criteria. Therefore, there was no clinical reason for giving infants liquids other than breastmilk during their neonatal hospital stay.

In our study the most commonly given liquid during hospital stay was formula milk (81%), followed by milk from the bank (11%) and sweetened water (dextrose solution, 8%). The inadequate practice of giving newborns formula milk in hospitals has also been documented in other parts of Mexico, both urban and rural (13), as well as in other countries (48–50). Giving newborns dextrose solution while in the hospital has been described as a common practice in Mexican rural communities since the late 1980's (51). Unfortunately, we did not ask the mothers the reasons why their infants were given liquids. It would be important to document the prevalence of such practices and their justifications not only among mothers but also among medical and nursing staff as well, particularly in hospitals that may not comply entirely with international guidelines such as WHO's Baby-friendly Hospital Initiative.

Women with larger bodies (i.e., higher BMI) at the time of the study visit were less likely to practice EBF beyond 1 month, a finding in accord with previous reports (52, 53). Since higher maternal BMI or higher fat mass percentage have been associated with shorter duration of EBF and delayed lactogenesis (53), physical, hormonal or socio-cultural factors are commonly proposed to explain such correlations (54). Physical concerns are usually about women with large breasts having difficulty adopting an adequate BF posture (55). Hormonally, women with large bodies have a lower prolactin response to suckling in the 1st days postpartum, but by day 7 their response is not different from those with smaller bodies, and serum progesterone diminishes equally in all of them (56). Finally, women with large bodies/breasts may have socially related concerns or body image problems that could limit their intention to BF (57).

But none of these interpretations for the association between shorter duration of BF and maternal BMI (breast size, adiposity, body image) holds true only for women with large bodies. Indeed, women who fall into the “normal” BMI category and have large breasts may face the same challenges with BF due to posture difficulties or delayed lactogenesis than women with larger bodies. Similarly, women who may be classified as having an adequate or even low BMI but who have body image problems may also choose not to BF or do so for shorter periods. However, in none of these cases would the women's body size (BMI) would be considered a barrier nor would they be encouraged to lose weight or diminish their weight gain, which is the traditional approach for women with larger bodies who are classified as overweight/obese based on BMI.

Furthermore, body size has also been shown to be related with socioeconomic conditions, both at the individual and country levels. In some social settings, larger bodies may reflect wealth or higher social status as body size is differentially related with various dimensions of individual socioeconomic level (e.g., income, education) as well as with country's level of development (58). Also, providing infants with milk substitutes (e.g., formula milk) is associated with wealth and may be considered a desired social trait (59).

When such a complex interrelation of bio-psycho-social factors related to body size is taken into account, it becomes clear that maternal body size (i.e., BMI) in itself may not be the true barrier to EBF. Therefore, in order to promote EBF, the rather simplistic focus on maternal BMI and weight reduction would not be an adequate approach and may only be reflecting weight stigma (60). Research is needed in order to distinguish, for example, between obesity and large breasts as separate challenges for breastfeeding (61). Maternal body size should not become a barrier for practicing EBF, when women with large bodies are provided with adequate, compassionate, non-biased counseling and accompanying, especially in the 1st days postpartum (55, 61, 62).

Similar to our results, several studies have documented that delivery mode influences breastfeeding rates (63). For example, a study held in China documented that infants delivered by C-section had lower breastfeeding rates from 1 to 6 months and were more likely to receive formula milk (64). However, when other variables concerning early feeding difficulties were considered, such as delayed initiation and weak suction consequence of anesthesia, the effect of C-section was attenuated or disappeared. Authors concluded that C-section *per se* was not a negative factor, but rather the difficulties that arise after the procedure. It should be noted that such difficulties are susceptible to breastfeeding counseling in order to reverse their negative effect on BF.

With regard to early postpartum, breastfeeding factors and beliefs, it is striking that, despite the fact that most of the women in our study did return to CESSA, FLHU, or other health facilities more than once during early postpartum, few reported receiving information and support regarding breastfeeding and some recalled receiving incorrect information. This has already been documented in a previous study in low-resource urban and rural populations from the Mexican states of Queretaro and Oaxaca (65). Indeed, participants who attended health facilities three or more times during early postpartum were more likely to practice >1 m-EBF. This could be interpreted in different ways that unfortunately we were not able to explore further due to our study design: women may have received more information and/or counseling during such visits; or they may comprise a highly motivated subgroup; or they may have easier access to health centers (e.g., living closer).

Another important thing to note is the fact that many women also attend private clinics and pharmacies when their child is ill, where they too receive infant feeding information. It has been documented both in Mexico and other countries that the promotion of breastmilk substitutes is a common practice in such facilities (8, 66, 67). For example, in a study in the Mexican states of Puebla and Chihuahua, authors documented that, when attending public and private health facilities, in 48.4% and 40.7% of cases, respectively, mothers of children younger than 24 months were recommended to give them a breast milk substitute. Researchers also found advertisements, discounted prices, promotional items, and even free samples for these foods in pharmacies (8).

After leaving the hospital, most participants in our study practiced on demand breastfeeding. However, more women in the ≤ 1 m-EBF group reported limiting the duration of the feeding, having latching difficulties, experiencing breast or nipple pain, and using a pacifier. These practices have been reported to reduce the duration of EBF (68, 69). Additionally, while maternal/infant illness has also been reported as a barrier for EBF (70, 71), it is important to note that the majority of participants in our study who stopped BF due to some illness, resumed the practice upon recovery.

After considering all the analyzed factors and their relationships, we were able to propose a conceptual model that illustrates their association with the duration of exclusive breastfeeding. The model (Figure 6) distinguishes between non-modifiable factors, which are represented in the upper part, that comprise situations mostly out of the woman's control. However, other factors result from the mother's immediate practices and beliefs; these are represented in the lower half of the conceptual model. Such factors would be central to the design of community-tailored interventions since they can be modified through appropriate information and counseling. It should be noted that women who present the non-modifiable factors should also receive close monitoring and counseling in order to overcome the intrinsic difficulties that would come with presenting those factors.

As stated above, the correct type and amount of information must be provided at prenatal care facilities to mothers and members of their inner social network alike, while ensuring its adequate comprehension and integration. During hospitalization and the 1st days/weeks after delivery, assertive personal counseling and accompaniment must be provided to mothers, irrespective of their individual conditions (e.g., previous BF experience, body size). And both information and counseling should be reinforced during the early postpartum, whether at health facilities or other settings. The higher aim is to translate this knowledge and awareness into more effective interventions that, when tailored to specific socio-cultural contexts, prove effective for increasing the duration of EBF.

### Study Limitations and Strengths

There are some limitations to our study. Since we did not use the WHO EBF indicator which asks about feeding practices the day prior to the interview and because our study has a cross-sectional design, several limitations arise: (1) caution must be used when comparing our information to others obtained using the WHO EBF indicator; (2) we cannot make causal claims about the relationship of the studied factors and EBF; and (3) we must consider a possible recall bias, specifically about the precise moment in which participants stopped EBF. However, regarding the latter, estimates of breastfeeding duration by maternal recall have been found to be reliable and valid during the first 3 years of the child's life (72).

It must also be kept in mind that we defined in our study >1 m-EBF as breastfeeding > 1 month postpartum, but this was defined in dependence upon our study population's practices and by no means can be considered long breastfeeding practice according to international recommendations.

Furthermore, due to the study design and inclusion criteria, our sample of participants had low risk of abandoning BF and was quite homogeneous in their sociodemographic characteristics. Thus, our results cannot be generalized to populations living in other socioeconomic settings or circumstances. However, it can give an idea of what happens with similar populations, which are of low-income, semi-rural and beneficiaries to the most basic government social security.

A strength of our study is that the sample showed a very similar prevalence of food insecurity as that reported for México. Data from the 2016 national health and nutrition survey documented that 70% of the population classified as having some degree of food insecurity, and that 29.5% classified in the categories of moderate and severe food security (14). Another similarity is the proportion of household welfare level distribution in our study, which was very similar to the percentages reported for all households in Tabasco during the year of the study (73).

## Data Availability Statement

The raw data supporting the conclusions of this article will be made available by the authors, without undue reservation.

## Ethics Statement

The studies involving human participants were reviewed and approved by Research and Ethics Committees of the National Institute of Perinatology in Mexico City. Written informed consent to participate in this study was provided by the participants' legal guardian/next of kin.

## Author Contributions

IV-O: investigation, methodology, data curation, and writing—review and editing. RV-S: formal analysis, writing—original draft, and review and editing. EM-M: investigation, data curation, and writing—review and editing. SH: conceptualization and writing—review and editing. MF-Q: conceptualization, methodology, formal analysis, supervision, writing—original draft, and review and editing. All authors contributed to the article and approved the submitted version.

## Funding

This study was partially funded by INPer, register 212250-3310-11406-03-16.

## Conflict of Interest

The authors declare that the research was conducted in the absence of any commercial or financial relationships that could be construed as a potential conflict of interest.

## Publisher's Note

All claims expressed in this article are solely those of the authors and do not necessarily represent those of their affiliated organizations, or those of the publisher, the editors and the reviewers. Any product that may be evaluated in this article, or claim that may be made by its manufacturer, is not guaranteed or endorsed by the publisher.
