# Supplementary material for: Exclusive Breastfeeding and Factors Influencing Its Abandonment During the 1st Month Postpartum Among Women From Semi-rural Communities in Southeast Mexico
Source: Front Pediatr. 2022 Feb 18;10:826295. doi: 10.3389/fped.2022.826295 (PMC8894443; doi:10.3389/fped.2022.826295)
Supplement: Supplementary file 1 [file Data_Sheet_1.PDF]

Supplementary Material. Exclusive breastfeeding questionnaire.

Section A: MOTHER'S ID AND SOCIODEMOGRAPHIC DATA
1. Name
2. Birth date
3. Age (years)
4. Address
5. Phone number
6. Do you live with your baby's father? ( ) yes ( ) no
7. What is your family type?
( ) Monoparental (you live alone with your baby and other children)
( ) Nuclear (You and your baby's father live together with you children)
( ) Extended with the mother's family (you and your partner live with your family)
( ) Extended with the father's family (you and your partner live with your partner's family)
8. What is your last completed school grade?
9. What is your current occupation?
( ) Student
( ) Housewife
( ) Employee, profesionist o businesswoman
( ) Other
10. Do you currently use any of these substances?
( ) yes ( ) no Cigarettes, how many a day?
( ) yes ( ) no Alcohol, times per week?
( ) yes ( ) no Other (specify)

Section B: LAST PREGNANCY
1. Was your last pregnancy planned? ( ) Yes ( ) No
2. Is this your first baby? ( ) yes ( ) no
(If yes, go to question 4)
3. How many pregnancies have you had? (including this baby)
4. How many of you babies were born alive?
5. During your last pregnancy, did you attend CESSA Villa Luis Gil Pérez or any of its medical units for prenatal care? ( ) yes ( ) no, How many appointments did you attend at CESSA Villa Luis Gil Pérez or any of its medical units? In what month of your pregnancy was the first visit?
6. Were you diagnosed with any of the following conditions during your last pregnancy?
a) Problems with blood sugar / Gestational diabetes ( ) yes ( ) no ( ) Don't know
b) Preeclampsia-eclampsia ( ) yes ( ) no ( ) Don't know
c) Depression ( ) yes ( ) no ( ) Don't know
d) Hypertension ( ) yes ( ) no ( ) Don't know
e) Another (specify)
7. Type of birth
( ) Vaginal
( ) Cesarean section
( ) Elective Cesarean section
( ) Emergency procedure
( ) Don't know
8. When was your last menstrual period? Date / Don't remember
9. When was your baby's birthdate? day/month/year
10. How many gestational weeks did your baby have at birth? Weeks / Don't know
11. What is your baby's sex?
12. What was your baby's birth weight? kilograms / Don't remember

13. What was your baby’s birth length? centimeters / Don’t remember

Section C: PREVIOUS BREASTFEEDING EXPERIENCE
If this is the woman’s first baby, go to the next section.

1. What is the birthdate of your previous child? day/month/year
2. How old is your previous child today? Years / Months
3. Did you ever breastfeed your previous child? ( ) Yes ( ) No
If Yes, go to question 7.
4. If not, for what reason(s) did you not breastfeed her/him?
5. How old was your previous child when you stopped breastfeeding her/him exclusively. In other words, when did you give her/him any food other than breastmilk (including water, formula milk, tea, cereals, juices or other food)? Months / Weeks / Don’t remember
6. How old was your previous child when you last breastfed her/him? In other words, the last time you nursed her/him. Years / Months / Weeks / Don’t remember
7. How did you feel about the experience of breastfeeding your previous child?
( ) Very unsatisfied ( ) Unsatisfied ( ) Indifferent ( ) Satisfied ( ) Very satisfied

Section D: BREASTFEEDING INFORMATION

1. During your prenatal care at CESSA or medical unit, did you receive information regarding the following subjects?
a) That you should exclusively breastfeed your baby. ( ) Yes ( ) No / For how long?
b) The optimal age for offering your baby food other than breastmilk.( ) Yes ( ) No / When?
c Which foods should be introduced first when starting complementary feeding. ( ) Yes ( ) No / Which ones?
2. Did you receive prenatal care somewhere else than CESSA or medical unit? ( ) Yes ( ) No
If yes,
2.1 Where?
2.2 From whom?
2.3 Did you receive information about breastfeeding? ( ) Yes ( ) No
2.4 Did you receive information about complementary feeding? ( ) Yes ( ) No
3. From what sources, other than CESSA or medical unit, did you receive information about breastfeeding?
( ) Yes ( ) No Radio
( ) Yes ( ) No Television
( ) Yes ( ) No Magazine
( ) Yes ( ) No Newspaper
( ) Yes ( ) No Other: ____
4. During your prenatal care at the medical unit or hospital, were you given any of the following?
( ) Yes ( ) No Formula milk
( ) Yes ( ) No Bottles
( ) Yes ( ) No Pacifiers
( ) Yes ( ) No Nipple shield or any device to help with breastfeeding
( ) Yes ( ) No Other items promoting any of the above
4.1 Where did you receive these?

Section E: AROUND THE BABY'S BIRTH

1. How long after your baby's birth did you breastfeed her/him for the first time? Minutes, Hours, Days ( ) Don't remember.
2. During your hospitalization, where was your baby?
( ) Your baby was always with you, day and night.
    How long after birth were you taken to your room?
( ) Your baby was not with you all the time.
    Why?
    Where was she/he?
3. During your hospitalization, did you or someone else give your baby something to drink other than your own breast milk? ( ) Yes ( ) No
If yes, what?
( ) Breast milk from the milk bank
( ) Formula milk
( ) Water
( ) Sweetened water
( ) Vitamins
( ) Other
( ) Don't know
4. During your hospitalization, was your baby given any liquid using a pacifier or bottle? ( ) Yes ( ) No ( ) Don't know
5. During your hospitalization, did you have any problem in your breasts or breastfeeding your baby? ( ) Yes ( ) No
If yes,
5.1 What problem?
5.2 How did you solve it?
6. After hospital discharge, were you breastfeeding your baby exclusively?
( ) Yes ( ) No ( ) Don't know
7. After hospital discharge, did you feel secure or insecure about being able to continue breastfeeding?
( ) Secure
( ) Insecure
8. During your prenatal care at the medical unit or hospital, were you given any of the following?
( ) Yes ( ) No Formula milk
( ) Yes ( ) No Bottles
( ) Yes ( ) No Pacifiers
( ) Yes ( ) No Nipple shield or any device to help with breastfeeding
( ) Yes ( ) No Other items promoting any of the above
8.1 Where did you receive these?
8.2 Who gave them to you?

Section F. CURRENT BREASTFEEDING EXPERIENCE AND COMPLEMENTARY FEEDING PRACTICES

1. After hospital discharge, were you breastfeeding your baby exclusively?
( ) Yes ( ) No ( ) Don't know
2. Since your last baby was born, how many times have you returned to CESSA or medical unit?
2.1 Why did you return?
2.2 During those visits to CESSA or medical unit, have you received support for breastfeeding or complementary feeding? ( ) Yes ( ) No
3. Since your baby was born, have you had any disease that prevented you from breastfeeding your baby? ( ) Yes ( ) No

3.1 Which one?
3.2 How did it affect you?
If you stopped breastfeeding then,
3.3 Did you resume breastfeeding? ( ) Yes ( ) No
4. Since your baby was born, have you seeked medical care for your baby anywhere other than CESSA or medical unit? ( ) Yes ( ) No
If yes,
4.1 Where?
4.2 Why?
4.3 Who attended you?
4.4 Did you receive support for breastfeeding or complementary feeding? ( ) Yes ( ) No
5. Are you still breastfeeding your baby? ( ) Yes ( ) No
If yes,
5.1 Do you breastfeed exclusively, that is, without giving formula milk, water, tea, juice, cereals or other food? (Note: in EBF, medicine and rehydration serum are allowed)
If yes, confirm,
5.2 So, you have never given your baby formula milk, water, tea, etc.? ( ) Yes ( ) No
If no,
5.3. For how long do you plan to continue giving your baby breastmilk exclusively?
If still breastfeeding but NOT exclusively,
5.4. How old was your baby when you stopped breastfeeding her/him exclusively (that is, without giving formula milk, water, tea, juice, cereals or other food? Months / Weeks
If NOT breastfeeding any more,
5.5 How old was your baby when you stopped breastfeeding her/him completely? Months / Weeks
6. During your current breastfeeding experience, do/did you have discomfort in your breast or nipples? ( ) Yes ( ) No
If yes,
6.1 What kind of discomfort?
6.2 How did you manage it?
6.3 Who helps/helped you?
7. During your current breastfeeding experience, has your baby had any difficulties holding on to your breast or sucking? ( ) Yes ( ) No
If yes,
7.1. What difficulty?
7.2. How did you manage it?
7.3. Who helps/helped you?
8. During your prenatal care at CESSA or medical unit, were you told you could return there for assistance in case of having breastfeeding problems? ( ) Yes ( ) No
9. Do/did you breastfeed your baby every time she/he asks for it without a schedule or do/did you follow a predetermined schedule?
( ) Free demand ( ) predetermined schedule ( ) Don’t know
If the answer was predetermined schedule,
9.1. How often do/did you breastfeed your baby?
10. Do/did you time how long you let the baby suck from each breast? ( ) Yes ( ) No
If yes,
10.1. How long do/did you let the baby suck from each breast? Minutes
10.2 How do/did you know when to switch breasts?
( ) You feel the breast empty.
( ) Your baby lets go of the breast.
( ) You time each take.
( ) You feel pain in your breast.

( ) Milk comes out of the other breast.
( ) Other
11. Does your baby use a pacifier? ( ) Yes ( ) No
11.1 When does she/he use it?
11.2 How old was she/he when you first gave it to her/him?
